# Analysis of Chinese patients with sporadic Creutzfeldt-Jakob disease

**DOI:** 10.1080/19336896.2020.1761515

**Published:** 2020-05-07

**Authors:** Jing Yang, Haiyan Kuang, Qiong Wang, Jiao Liu, Xueping Chen, Huifang Shang

**Affiliations:** a Department of Neurology, West China Hospital, Sichuan University, Chengdu, Sichuan, China; b Department of Neurology, The Second People’s Hospital of Banan District, Chongqing, China; c Neurological Intensive Care Unit, West China Hospital, Sichuan University, Chengdu, Sichuan, China

**Keywords:** Sporadic Creutzfeldt-Jakob disease, clinical manifestation, survival, a tertiary care hospital, literature review

## Abstract

Sporadic Creutzfeldt-Jakob disease (sCJD) is a rare, incurable, and fatal neurodegenerative disorder. The objective of this study was to describe the clinical features and survival time of Chinese sCJD patients, and to explore the associations between clinical data and survival. In this study, we analysed the clinical data of 21 sCJD patients in a tertiary care hospital and used all Chinese case material available from 152 patients with sCJD in literatures between 2008 and 2018. The mean age of onset of all 173 deceased patients was 61.44 year-olds (y), with the highest incidence in the population of 60 to 69 y. The most common manifestation at disease onset was progressive dementia. With the progression of the disease, the four main clinical symptoms and signs were developed, including myoclonus, visual or cerebella disturbance, pyramidal or extrapyramidal dysfunction, and akinetic mutism. Extrapyramidal symptoms were more frequently observed. The mean survival time was 7.34 months, and 82.10% of cases died within 1 year after disease onset. The follow-up showed that the survival time was longer and the myoclonus sign was more frequently presented in younger-onset sCJD patients. Patients with abnormalities only in cortical regions had a higher frequency of pyramidal dysfunction than patients having lesions in both cortex and basal ganglia. The findings of this study might provide some insight into the clinical characteristics of sCJD patients in China, but further studies could examine the presences of clinical features and survival time in patients with early age of onset in a prospective manner.

## Introduction

Creutzfeldt-Jakob disease (CJD) is a rare, incurable, and inevitably fatal neurodegenerative disorder [1]. The pathophysiological mechanism of CJD consists of the formation of an abnormal isoform of prion protein (PrP) called scrapie prion protein (PrPSc), and the accumulation of these abnormal proteins disrupts cell function and causes a sponge-like appearance in brain tissue [2,3]. CJD has a worldwide distribution, and the estimated incidence in the United States is 0.5 to 1 per million inhabitants per year [4,5]. There are four subtypes of CJD: sporadic (sCJD), familial (fCJD), iatrogenic (iCJD) and variant CJD (vCJD). sCJD is the most common form, accounting for ~85% of all cases. Clinical presentation of sCJD is highly variable; symptoms can range from rapidly progressive dementia to neuropsychiatric manifestations, cerebellar ataxia, visual impairment, akinetic mutism, myoclonus, and pyramidal and/or extrapyramidal signs [6]. The diagnosis of sCJD can be challenging due to the variegated symptoms and signs. To facilitate diagnosis, paraclinical tests, including electroencephalogram (EEG), cerebrospinal fluid (CSF) biomarkers, magnetic resonance imaging (MRI) of the brain, positive real-time quaking-induced conversion (RT-QuIC) and brain biopsy, can be used.

Here, we investigated the clinical characteristics and survival time of the deceased Chinese sCJD patients, including a case series in a tertiary care hospital and all the Chinese sCJD cases reported in medical journals, trying to address the associations between clinical characteristics and survival time.

## Patients and methods

In the tertiary care hospital-West China Hospital of Sichuan University, the suspected CJD cases were diagnosed using the diagnostic criteria for CJD issued by the China National Health Commission [6]. The clinical data, including the general information of the patient, main clinical manifestations, the typical symptom and results of paraclinical tests were recorded in detail. The appearance of periodic sharp wave complexes (PSWC) was regarded as a specific EEG abnormality. The presence of high signal intensities in the caudate/putamen and/or cortical ribboning in diffusion-weighted imaging (DWI) was considered to be abnormal. Western blots for 14-3-3 in the CSF, and PRNP PCR and sequencing were also performed. The first presentation of neurological signs or symptoms suggestive of organic involvement was judged as disease onset. The manifestation of lacking voluntary movement and the ability to produce meaningful words were defined as akinetic mutism state [7]. All patients were followed up by telephone or face-to-face interview with an interval of 3 months. The survival time was defined as the duration from disease onset to death. All the participants gave their written informed consents; the Ethical Committee of West China Hospital approved this study. The electronic databases Chinese National Knowledge Infrastructure and Wanfang (Chinese) were utilized to search for terms including ‘Creutzfeldt-Jakob disease’ or ‘prion disease’. We also conducted a search on PubMed, restricting the search to studies conducted in China. To avoid missing literatures, we also looked through the references of relevant articles; aiming to collect all the studies regarding the Chinese sCJD cases reported within the last 10 years up until December 2018. Studies which reported the clinical data and survival time were retained for this study. Comparisons of continuous variables between two groups were made using Student’s t-test. Categorical variables were compared using Fisher’s exact test. All data were presented in the form of mean ± standard deviation, and they were analysed using SPSS 17.0. A p-value of less than 0.05 was considered to be statistically significant.

## Results

### General demographic features

Between 1 January 2008 and 31 December 2018, 21 patients were diagnosed as probable sCJD in Sichuan University West China Hospital according to the diagnostic criteria for sCJD; 7 were males and 14 were females, with a gender ratio of 0.5:1. The disease onset age ranged from 47 to 81 year-old (y), and the mean age of onset was 62.67 y. The highest incidence in our cohort was 60 to 69 y, and ten patients (47.62%) had an onset of illness between 60 and 69 years of age, 90.48% of cases were older than 50 years. All the patients were deceased during follow-up, and the medium survival time was 5.48 months. The clinic manifestations of these 21 sCJD patients were listed in supplementary Table 1. Between 2000 and 2018, data from articles published in China or abroad showed that 152 patients were diagnosed as probable sCJD according to the diagnostic criteria for sCJD, and the information of included studies (publish date, journal, and authors) ﻿are listed in supplementary Table 2. Out of the reported sCJD patients, 74 were males and 78 were females, with a gender ratio of 0.95:1. The disease onset age varied from 18 to 83 y, with the mean age of onset of 61.27 y. The majority of patients (63.82%) had an onset of disease between 50 and 69 years of age (52 cases were in the 60–69 y group, 45 cases were in the 50–59 y group), and 88.16% of patients were older than 50 years. The medium survival time for all these deceased patients was 7.34 months. The demographics of all included sCJD patients differentiated by gender and age of onset are listed in Table 1.

**Table 1.** Demographics of Chinese patients with sCJD differentiated by gender and age of onset.

		Gender			Age of onset		
Item	Total	Male	Female	*P value*	≤50 y	>50 y	*P value*
Number	173	81	92	-	21	152	
Age of onset (y)	61.44 ± 10.42	61.63 ± 10.73	61.27 ± 10.29	0.8223	-	-	-
Survival time (m)	7.34 ± 7.57	6.86 ± 9.54	7.78 ± 5.21	0.4653	11.88 ± 8.90	6.67 ± 7.15	**0.0030**

## Clinical features

Most of the patients displayed more than one symptom at disease onset. Among the presenting symptoms, progressive memory loss and cognitive dysfunction were mostly described, which appeared in 42.28% cases. Mental and behaviour disorder, including depression, anxiety, apathy, impediment speech irritability, illusion, emotional lability and personality changes, was reported in 19.46% cases. Ataxia and gait instability were recorded in 16.11% cases, followed by visual disturbances which appeared in 10.07% cases. Nine patients showed extrapyramidal symptoms (including slowness, stiffness, tremors, and involuntary movement), and two patients exhibited pyramidal symptoms or signs. Additionally, there was one patient who was first presenting with stroke and one patient who had myoclonus as an initial symptom. However, 15 patients (8.67%) showed atypical clinical manifestations at disease onset; they only complained about dizziness at first.

With the progression of disease, more symptoms and signs were identified. Progressive dementia was noticed in 100% of patients. Extrapyramidal symptoms (77.42%) also appeared frequently, followed by myoclonus (63.64%), pyramidal symptoms (60%), cerebellar problems (54%) and akinetic mutism (48.24%). Visual disturbance was reported in 40.58% cases. The differences in the clinical major manifestations were further investigated in all included sCJD cases, who were subgrouped by gender, age of onset (≤50 y vs >50 y), DWI imaging (only cortical regions vs both cortical regions and basal ganglia), and survival time (≤6 m vs >6 m). Except for dementia, the presence of other major symptoms of sCJD was compared. No statistical differences in the presences of main manifestations were found in patients with different gender and survival time. However, the presence of myoclonus in patients with different onset age showed significantly different tendencies. The positive rate of myoclonus in sCJD patients with younger-onset (78.05%) was significantly higher than that in patients with older-onset (60.56%). Besides, pyramidal dysfunction was noted comparably in sCJD patients with different imaging manifestations on DWI; the presence of pyramidal dysfunction was more frequently in patients who had abnormalities only in cortical regions (72.22%), compared to patients who had lesions in both cortical regions and basal ganglia (46.68%). The comparison of the major symptoms among subgroups is shown in Table 2.

**Table 2.** The subgroup analysis of the presence of clinical features during the course of sCJD.

	Gender			Age of onset			survival			DWI Image		
Item	Male	Female	*P value*	≤50 y	>50 y	*P value*	≤6 m	>6 m	*P value*	Cortex	Cortex & basal ganglia	*P value*
Myoclonus	50/75 (66.67%)	48/79 (60.76%)	*0.5041*	26/33 (78.79%)	84/140 (60%)	***0.0468***	60/98 (61.22%)	38/56 (67.86%)	*0.4871*	25/40 (62.50%)	34/53 (64.15%)	*1*
Cerebella disturbance	44/73 (60.27%)	37/77 (48.05%)	*0.1437*	15/26 (56.69%)	78/147 (53.06%)	*0.8433*	55/102 (53.92%)	28/50 (56%)	*0.8633*	23/36 (63.89%)	30/47 (63.83%)	*1*
Pyramidal dysfunction	37/65 (56.92%)	38/60 (63.33%)	*0.5838*	20/34 (58.82%)	94/150 (62.67%)	*0.6989*	51/84 (60.71%)	24/41 (58.54%)	*0.8475*	26/36 (72.22%)	21/47 (44.68%)	***0.0148***
Extrapyramidal dysfunction	49/65 (75.38%)	47/59 (79.66%)	*0.6687*	22/28 (78.57%)	115/146 (78.77%)	*1*	63/83 (75.90%)	33/41 (80.49%)	*0.6521*	27/36 (75%)	38/46 (82.61%)	*0.4239*
Visual disturbance	30/68 (44.12%)	26/70 (37.14%)	*0.4884*	15/31 (48.39%)	58/139 (41.73%)	*0.5729*	38/92 (41.30%)	18/46 (39.13%)	*0.8555*	17/36 (47.22%)	21/47 (44.68%)	*0.8281*
Akinetic mutism	26/56 (46.43%)	29/58 (50%)	*0.7124*	12/29 (41.38%)	75/152 (49.34%)	*0.5437*	34/74 (45.95%)	19/38 (50%)	*0.6949*	20/34 (58.82%)	20/46 (43.48%)	*0.2580*

Significant values are highlighted in bold characters.

## Survival time

All included cases in the present study had died on a known date. The mean survival time of all included cases was 7.34 months, with a range of 0.5 to 72. The cumulative incidences of the survival time which was less than 3, 6, 12 and 24 months were 14.20%, 53.09%, 82.10% and 97.53%, respectively. As shown in Table 1, younger-onset patients (onset age ≤50 y) had longer survival time than patients with older-onset (onset age >50 y), but no statistical difference in survival time was found between two genders. All the included patients were examined by DWI imaging; patients with only cortical regions abnormalities had similar survival time to those patients who had both cortical regions and basal ganglia affected. The association between the survival time and the frequency of the major manifestations was analysed, and the main symptoms of progressive dementia, myoclonus, visual disturbance, cerebella disturbance, pyramidal dysfunction, extrapyramidal dysfunction, and akinetic mutism were evaluated in all the included patients. The results showed that the survival time of the patients with more than three signs (>4) were not significantly different from those with less than three signs (≤3).

## Discussion

In the current study, we systematically describe the clinical features and survival time of 173 sCJD cases, among them 21 patients were from West China Hospital-a tertiary care hospital in China. Since the number of sCJD patients in a third-level hospital was too small to analyse the associations between clinical features and survival time, we did a literature search to include all the reported sCJD cases or case series from China. All the included sCJD patients were deceased, and the survival time was recorded.

There were more female than male sCJD patients in the current investigation, and the male to female ratio was 0.88/1. Similarly, a preponderance of female cases was also reported in most previous studies [8–14]. However, a surveillance study from China found more males in 261 sCJD patients and a male to female ratio of 1.27:1 [15]. In addition, a previous review stated that sCJD occurs equally in both sexes [16]. Furthermore, the survival time seemed to be longer in females, but the difference was not significant. Another Japanese study also found that disease duration was longer in females, but this tendency was not statistically significant [17]. Therefore, the gender differences in survival time could not reach an agreed conclusion and more reliable foreign data are required in future studies. The mean onset age was 61.44 years (range: 18–82) in the present study. Similarly, another Chinese study found a median onset age of 61 years [15], and a meta-analysis showed that the mean age of onset was 60.7 years in 3083 sCJD patients [18]. The data from our hospital and the literature both showed that the majority of cases had an onset of disease between 60 and 69 years of age, with a 25%/75% percentile value of 56/70. This finding was consistent with previous studies, which reported a peak age of onset between 60 and 69 years [15,16,19]. Only 21 sCJD patients were under 50 years old at disease onset, and we divided our sCJD patients into two groups using the cut-off value of 50 years. The results showed that patients with younger-onset (≤50 y) had a significantly longer survival time than patients with older-onset (>50 y). A similar study divided the 674 sCJD patients into four groups according to the onset age (≤39, 40–59, 60–79, ≥80 y), and they found that earlier disease onset may be associated with longer survival time, and the probabilities were 0.071 for age group [17]. A study from Germany also found that younger patients (≤50 y) with sCJD had significantly longer survival time than older patients (>50 y) [20]. Young age at disease onset was demonstrated to be one of the major factors attributing to the survival of CJD [21]. Since the clinical, pathological, and laboratory features of sCJD patients with younger-onset are quite different from those of older-onset cases [22], younger onset age could be related to a better prognosis, but this trend requires further validation by studies with larger sample size.

Progressive memory loss and cognitive dysfunction were most detectable presenting symptoms, followed by mental disorder, cerebellum symptoms. The frequencies of presenting symptoms in our sCJD patients were similar to those reported in a previous study [15]. We also noticed that 8.67% of sCJD patients experienced dizziness as the presenting symptom, and this kind of symptoms is non-specific and easily misdiagnosed. Dizziness presentation is not frequent in sCJD, which was reported to be present as an initial symptom in 2.6% of CJD patients [18]. Studies also reported that the initial symptoms of CJD could mimic benign peripheral vestibulopathy, leading to a misdiagnosis or a delayed diagnosis [23–26]. The relatively high percentage of dizziness as an initial manifestation in Chinese sCJD patients may due to the lack of a thorough knowledge of early deficits of sCJD, and it is important to raise awareness of the clinical symptoms and presentations of this rare disease. As the disease progressed to a more advanced stage, more symptoms had been observed. There was no significant difference in terms of the main symptoms, when our sCJD patients were divided into two groups by gender and survival time. However, when they were grouped by onset age, myoclonus was more frequently released in our patients with younger-onset (≤50 y), compared to those patients with older-onset (>50 y). Relatively high frequency of myoclonus sign (90%) in young sCJD patients during disease course had been reported previously [20]. The frequency of visual or cerebellar disturbance in young sCJD patients was reported to be lower than that of total sCJD [22], but we did not find such pattern. In the present study, the positive rate of visual and cerebellar disturbance was 48.57% and 56.67% in sCJD patients with young-onset, comparable with the results from all sCJD patients (43.15% and 53.69%, respectively). In addition, the frequency pyramidal dysfunction was higher in patients with abnormalities only in cortical regions compared to those patients who had lesions in both cortical regions and basal ganglia. Pathologically in sCJD, the most severely affected region is neocortex, and the severity of lesion is related to the disease duration [27,28]. The previous study found that abnormalities of the only cortical regions were found in 24% of sCJD patients, while the alterations in the basal ganglia region and the presence of cortical ribboning were present in 68% [29]. Therefore, along with the progression of disease, pyramidal dysfunction is observed, and the combination with the hyperintensity in the cerebral cortex in DWI image may indicate the diagnosis of the sCJD, even though there is no DWI hyperintensity in basal ganglia region. However, we should be concerned about the bias produced by the limited sample size; further study with more case numbers is required. We also analysed the association of survival time with the appearance of the main manifestations in our sCJD patients during disease progression and found that patients who had more than three signs had similar survival time with those patients who had less than three neurological manifestations. A large portion of sCJD patients had more than three manifestations during the disease progression, but no matter how many main manifestations were present, there were no statistical differences in the survival time. It is possible that when more than three manifestations are presented, an extended neuropathologic damage has already occurred in sCJD patients.

The mean survival time of the present study was 7.34 months, with a range of 0.5–72; and 82.10% of patients died within 1 year after onset. Similarly, the Chinese surveillance data from 2008 to 2011 showed a mean survival time of 6.1 months and a 1-year mortality of 74% [30]. The data from China are comparable to that of Western countries. The European CJD Surveillance Network (EuroCJD) included 2,451 deceased sCJD patients from 31 December 1992 to 31 December 2002 and found a median survival time of 5 months (range: 1–81) and a 1-year mortality of 85.8% [14]. The surveillance of CJD in Argentina (1997–2008) also revealed that the mean survival time was 4.6 months (range: 1–70) in sCJD patients [31]. However, the Japanese CJD surveillance programme from 1 April 1999 through 4 September 2008 reported a longer survival time of 17.4 months in patients with prion diseases, and 46.0% patients died within 1 year after disease onset. The inconsistent findings from Japanese study are likely resulted from the country’s healthcare system [17]. An observational study indicated that sCJD patients from North American and European died shortly after reaching the akinetic mutism state [32]. This phenomenon was probably due to financial and ethical concerns. In Western countries and China, patients with fatal neurological disorders normally did not receive the intensive life-sustaining treatments, but the tube-feeding and mechanical ventilation therapy were commonly implemented for Japanese sCJD patients even they had reached the akinetic mutism state [33].

There are several limitations to this study. Only 21 sCJD patients were from our hospital, a large portion of patients was gleaned from the literature, and the quality of the study was relying on the data of the literature. Some case reports may specifically report the atypical sCJD patients, which would cause bias. Additionally, some studies did not conduct the PRNP analysis; it is possible that some cases could actually be fCJD cases.

In summary, this study showed the clinical manifestations and survival time of Chinese sCJD patients, and the differences in the survival time between younger-onset and older-onset patients. With the development of the clinical syndrome, younger-onset patients more frequently showed myoclonus sign, and patients having abnormalities only in cortical regions had a higher frequency of pyramidal dysfunction than patients with lesions in both cortex and basal ganglia. The high frequency of myoclonus and prolonged survival time in younger-onset sCJD patients might have some implications for clinical practice, and further studies could examine the presences of clinical features and survival time in patients with different ages of onset in a prospective manner.
