# Supplementary material for: Analysis of Chinese patients with sporadic Creutzfeldt-Jakob disease
Source: Prion. 2020 May 7;14(1):137–42. doi: 10.1080/19336896.2020.1761515 (PMC7219432; doi:10.1080/19336896.2020.1761515)
Supplement: Supplemental Material [file kprn-14-01-1761515-s001.docx]

Table 1. The clinical manifestations of 21 sCJD patients in a tertiary care hospital

| Case No | gender | Onset of age | dementia | myoclonus | visual disturbance | cerebella disturbance | pyramidal dysfunction | extrapyramidal dysfunction | akinetic mutism | EEG | CSF analysis 14‑3‑3 protein | MRI imaging | Median survival time (m) |
| --- | --- | --- | --- | --- | --- | --- | --- | --- | --- | --- | --- | --- | --- |
| 1 | male | 58 | + | - | - | + | - | - | + | slow waves of background activity | - | 3 | 7 |
| 2 | male | 51 | + | + | - | + | - | + | + | slow biphasic waves | + | 2 | 6 |
| 3 | female | 67 | + | - | + | - | - | + | - | PSWC | - | 2 | 9 |
| 4 | female | 62 | + | - | - | + | + | + | - | slow waves of background activity | - | 2 | 5 |
| 5 | female | 81 | + | + | + | - | - | - | + | PSWC | + | 2 | 4 |
| 6 | female | 71 | + | + | + | + | - | + | - | PSWC | + | 2 | 4 |
| 7 | male | 64 | + | + | - | + | - | + | + | PSWC | - | 3 | 9 |
| 8 | female | 52 | + | - | - | - | + | + | + | PSWC | + | 3 | 5 |
| 9 | female | 58 | + | + | - | - | + | + | + | PSWC | + | 2 | 6 |
| 10 | female | 47 | + | - | + | + | + | - | - | slow biphasic waves | + | 3 | 7 |
| 11 | female | 64 | + | - | - | - | - | + | + | PSWC |  | 3 | 6 |
| 12 | female | 73 | + | - | - | + | + | + | + | slow waves of background activity | + | 3 | 5 |
| 13 | male | 62 | + | + | - | + | - | + | + | PSWC | + | 3 | 3 |
| 14 | male | 63 | + | + | + | - | - | - | - | PSWC | + | 2 | 3 |
| 15 | female | 66 | + | + | - | + | - | + | + | PSWC | + | 3 | 3 |
| 16 | male | 56 | + | + | - | + | - | - | - | slow waves of background | - | 1 | 8 |
| 17 | female | 66 | + | - | - | + | + | + | + | PSWC | + | 3 | 8 |
| 18 | female | 63 | + | - | - | - | + | + | + | slow biphasic waves | - | 1 | 6 |
| 19 | female | 48 | + | - | + | - | + | - | + | PSWC | + | 2 | 3 |
| 20 | male | 77 | + | + | - | - | + | - | + | slow waves of background | - | 3 | 3 |
| 21 | female | 67 | + | + | + | + | - | + | + | PSWC | + | 3 | 5 |

| Abbreviations: +:positive; -: negative; PSWC: periodic sharp wave complexes; MRI imaging 1: hyperintensity in the caudate nucleus/putamen on DWI sequence; 2: hyperintensity in ≥ 2 cortical regions on DWI sequence; 3: hyperintensity in both the caudate nucleus/putamen and ≥ 2 cortical regions on DWI sequence. |
| --- |

Table 2. The general information of included studies

| Publish date | Journal | Authors | Reference |
| --- | --- | --- | --- |
| 2019/6/1 | 吉林大学硕士学位论文 | 戴妍源 | [1]戴妍源. 散发型克雅氏病患者临床表现及多导睡眠监测（PSG）特点分析[D].: 吉林大学, 2019. |
| 2019/10/1 | 当代护士 | 杨俊行 胡哲 苏荣 | [2]杨俊行,胡哲,苏容. 2例克雅病患者的临床观察与护理[J]. 当代护士(下旬刊), 2019, (10): 37-39. |
| 2019/6/15 | 中华实验和临床感染病杂志 | 吴雅丽;伍文清;黄宇明;许东梅;姜美娟;马小扬; | [3]吴雅丽,伍文清,黄宇明,等. 六例散发型克雅病患者临床分析[J]. 中华实验和临床感染病杂志(电子版), 2019, 13(3): 255-259. |
| 2019/2/26 | 卒中与神经疾病 | 潘合跃;徐建洋;李洪亮;卢斯猛;王晓华;史向松;王守勇; | [4]潘合跃,徐建洋,李洪亮,等. 3例Creutzfeldt-Jakob病患者的临床特点分析并文献复习[J]. 卒中与神经疾病, 2019, 26(1): 105-108. |
| 2018/1/5 | 临床荟萃 | 尹阔场;罗欣彤;赵立明;张海宁;马如雪;檀国军; | [5]尹阔场,罗欣彤,赵立明,等. 散发型克雅病1例并文献复习[J]. 临床荟萃, 2018, 33(1): 82-83, 86. |
| 2018/4/6 | 世界最新医学信息文摘 | 许国安;王跃彬;孙佳; | [6]许国安,王跃彬,孙佳. 以视力减退为首发症状的克雅氏病1例及文献复习[J]. 世界最新医学信息文摘, 2018, 18(28): 205-206. |
| 2018/2/28 | 中风与神经疾病杂志 | 赵名娟;陈泽;邹越华;周翠玲;程洁; | [7]赵名娟,陈泽,邹越华,等. Creutzfeldt-Jakob病1例报告并文献复习[J]. 中风与神经疾病杂志, 2018, 35(2): 166-167. |
| 2018/3/20 | 中国病毒病杂志 | 何桂香; | [8]何桂香. 5例散发型克雅病临床诊断病例的特征研究[J]. 中国病毒病杂志, 2018, 8(2): 151-155. |
| 2018/7/25 | 中国实验诊断学 | 戴妍源;吕洋;郎悦;吕晓民; | [9]戴妍源,吕洋,郎悦,等. 散发型克-雅氏病2例典型脑电图分析并文献回顾[J]. 中国实验诊断学, 2018, 22(7): 1155-1157. |
| 2017/10/15 | 临床军医杂志 | 关彩萍;付学锋;田文静; | [10]关彩萍,付学锋,田文静. 克-雅氏病1例报道[J]. 临床军医杂志, 2017, 45(10): 1099-1100 |
| 2017/3/22 | 临床误诊误治 | 靳洲;马琳;李其富;陈蓉;王淑荣;陈志斌;廖小平; | [11]靳洲,马琳,李其富,等. 散发性Creutzfeldt-Jakob病一例误诊报告[J]. 临床误诊误治, 2017, 30(3): 34-36. |
| 2017/11/20 | 实用老年医学 | 王本孝; | [12]王本孝. 以进展性痴呆为表现的克雅病1例报道[J]. 实用老年医学, 2017, 31(11): 1099-1100. |
| 2017/6/26 | 卒中与神经疾病 | 郭方亮;胡社静;李涛; | [13]郭方亮,胡社静,李涛. 散发型克雅病7例患者的临床、脑电图及影像学分析[J]. 卒中与神经疾病, 2017, 24(3): 217-222 |
| 2016/10/25 | 临床神经病学杂志 | 陶雯;赵映红;倪耀辉;柯开富; | [14]陶雯,赵映红,倪耀辉,等. 散发型Creutzfeldt-Jakob病1例报告[J]. 临床神经病学杂志, 2016, 29(5): 397-399 |
| 2016/5/30 | 中风与神经疾病杂志 | 郎文娟;孙元元;李慧;王栋;崔俐;冯加纯; | [15]郎文娟,孙元元,李慧,等. 克-雅氏病5例临床分析[J]. 中风与神经疾病杂志, 2016, 33(5): 429-432. |
| 2016/2/27 | 中国神经精神疾病杂志 | 白静;孙云闯;孙葳;吕鹤; | [16]白静,孙云闯,孙葳,等. 伴瘙痒症状的Heidenhain变异型克雅氏病3例报告并文献复习[J]. 中国神经精神疾病杂志, 2016, 42(2): 96-99. |
| 2016/11/27 | 中国神经精神疾病杂志 | 吴萱;林艾羽;李智文;王志强;王柠; | [17]吴萱,林艾羽,李智文,等. 散发型克-雅病的临床特点分析[J]. 中国神经精神疾病杂志, 2016, 42(11): 676-679. |
| 2015/4/10 | 海南医学 | 李惠明;曹慧芳;黄琛;刘璐; | [18]李惠明,曹慧芳,黄琛,等. 散发型克雅氏病的MRI诊断价值[J]. 海南医学, 2015, 26(7): 992-994. |
| 2015/8/25 | 临床神经病学杂志 | 黄承芳;姜丹;金红花;张智燕;杨卫;吴银侠 | [19]黄承芳,姜丹,金红花,等. Creutzfeldt-Jakob病的MRI动态改变(附1例报告)[J]. 临床神经病学杂志, 2015, 28(4): 305-307. |
| 2015/5/30 | 中风与神经疾病杂志 | 宫淑杰;姚庆阳;王惠民;李植灿; | [20]宫淑杰,姚庆阳,王惠民,等. 临床诊断散发型克雅病3例并文献复习[J]. 中风与神经疾病杂志, 2015, 32(5): 464-466. |
| 2015/4/15 | 中国人兽共患病学报 | 周洋;林赟; | [21]周洋,林赟. 昆明市首例散发型克雅氏病临床诊断病例的发现与调查[J]. 中国人兽共患病学报, 2015, 31(4): 391-393. |
| 2015/6/27 | 中国神经精神疾病杂志 | 张尊胜;胡珍珠;李可;王永;李举;薛国松;祖洁; | [22]张尊胜,胡珍珠,李可,等. 散发性Creutzfeldt-Jakob病的头颅MRI特点与鉴别诊断[J]. 中国神经精神疾病杂志, 2015, 41(6): 331-335. |
| 2016/1/2 | 中国医学影像学杂志 | 郭喆;王瑞民; | [23]郭喆,王瑞民. 很可能的散发型Creutzfeldt-Jakob病脑葡萄糖代谢统计参数图分析[J]. 中国医学影像学杂志, 2015, 23(12): 884-887, 891. |
| 2014/9/18 | 中华医学会第十七次全国神经病学学术会议 | 张尊胜;张尊胜;李可;王永;李举;薛国松;祖洁 | [24]张尊胜,张尊胜,李可,等. 散发型Creutzfeldt-Jakob病的临床表现、脑电图及影像学特点, 2014. |
| 2014/10/30 | 中风与神经疾病杂志 | 卢禹 | [25]卢禹. Creutzfeldt-Jakob病的MRI表现[J]. 中风与神经疾病杂志, 2014, 31(10): 944-945. |
| 2013/4/12 | 山东大学硕士学位论文 | 吕园 | [26]吕园. 5例克雅氏病临床分析并文献复习[D].山东大学, 2013. |
| 2013/8/25 | 临床神经病学杂志 | 曹化;仲玲玲;孙波; | [27]曹化,仲玲玲,孙波. 脑电图和MR弥散加权成像对皮质-纹状体-脊髓变性的诊断价值(附3例报告)[J]. 临床神经病学杂志, 2013, 26(4): 309-311. |
| 2013/12/15 | 中华老年心血管病杂志 | 刘晓彩;王国强;张微微;李莹;高妹茹 | [28]刘晓彩,王国强,张微微,等. 脑卒中样发作为首发临床表现的克雅病三例[J]. 中华老年心脑血管病杂志, 2013, 15(12): 1329-1330. |
| 2013/9/20 | 现代电生理学杂志 | 李泽;李蒙燕;郑浩 | [29]李泽,李蒙燕,郑浩. 散发型Creutzfeldt-Jakob病的临床、脑电图及头颅磁共振DWI影像特点研究[J]. 现代电生理学杂志, 2013, 20(3): 131-137. |
| 2012/7/15 | 临床药物治疗杂志 | 母艳蕾;彭丹涛; | [30]母艳蕾,彭丹涛. 散发型克—雅病的临床特点探讨[J]. 临床药物治疗杂志, 2012, 10(4): 9-12. |
| 2012/5/30 | 中风与神经疾病杂志 | 张晓燕;邓方;宋晓南; | [31]张晓燕,邓方,宋晓南. 散发性基因变异性CJD2例报告及文献回顾[J]. 中风与神经疾病杂志, 2012, 29(5): 402-404. |
| 2012/8/30 | 中风与神经疾病杂志 | 曹笃;魏有东;李琦;谢鹏; | [32]曹笃,魏有东,李琦,等. 磁共振弥散加权对克-雅氏病早期诊断的意义[J]. 中风与神经疾病杂志, 2012, 29(8): 726-728. |
| 2011/2/28 | 河北医科大学学报 | 解冰川;解冰;顾平;王铭维; | [33]解冰川,解冰,顾平,等. 临床疑似克雅病误诊1例[J]. 河北医科大学学报, 2011, 32(2): 154, 248. |
| 2011/3/20 | 临床放射学杂志 | 潘仲林;朱友志;吴满丽;宋伟; | [34]潘仲林,朱友志,吴满丽,等. Creutzfeldt-Jakob病MRI诊断(附2例报道及文献复习)[J]. 临床放射学杂志, 2011, 30(3): 444-447. |
| 2011/6/15 | 中华老年心血管病杂志 | 吴杰贤;梁颖茵;姚晓黎 | [35]吴杰贤,梁颖茵,姚晓黎. 克雅病早期误诊临床分析[J]. 中华老年心脑血管病杂志, 2011, 13(6): 535-537. |
| 2011/4/25 | 齐鲁护理杂志 | 张海燕; | [36]张海燕. 散发型克雅氏病1例临床护理[J]. 齐鲁护理杂志, 2011, 17(12): 74-75. |
| 2011/8/30 | 中风与神经疾病杂志 | 王广文;李娜;吕玉丹;林卫红; | [37]王广文,李娜,吕玉丹,等. 动态脑电图观察对Creutzfeldt-Jakob病的诊断价值探讨(附1例临床报告)[J]. 中风与神经疾病杂志, 2011, 28(8): 755-756. |
| 2011/8/15 | 中国误诊学杂志 | 吕晓静;朱宗红; | [38]吕晓静,朱宗红. 克雅病1例的护理[J]. 中国误诊学杂志, 2011, 11(23): 5784. |
| 2010/12/25 | 神经病学与神经康复学杂志 | 杜奉舟;李颖; | [39]杜奉舟,李颖. 很可能的克雅病1例报道[J]. 神经病学与神经康复学杂志, 2010, 7(4): 223, 240. |
| 2010/8/27 | 中国神经精神疾病杂志 | 李洁;刘睿;李柱一;苗建亭; | [40]李洁,刘睿,李柱一,等. Creutzfeldt-Jakob病1例报告[J]. 中国神经精神疾病杂志, 2010, 36(8): 505-506. |
| 2008/3/15 | 蚌埠医学院学报 | 许力;陈齐鸣;韦道祥;刘晓林;吴守伟;胡明洁; | [41]许力,陈齐鸣,韦道祥,等. Creutzfeldt-Jakob病的临床诊断(附2例报道)[J]. 蚌埠医学院学报, 2008, (2): 155-157. |
| 2008/3/1 | 解放军医学杂志 | 王国强;尹维民;张微微; | [42]王国强,尹维民,张微微. 克雅氏病临床诊断1例[J]. 解放军医学杂志, 2008, (3): 358. |
| 2008/4/20 | 临床神经电生理学杂志 | 崔爱勤;徐家立;李扬波;陈玉平;刘玉玺; | [43]崔爱勤,徐家立,李扬波,等. 克罗伊茨费尔特-雅各布病1例临床与病理分析[J]. 临床神经电生理学杂志, 2008, (2): 126-127. |
| 2008/6/20 | 临床神经电生理学杂志 | 王锦玲;于如山;史雪颖;曲方;王丽萍;冯阳; | [44]王锦玲,于如山,史雪颖,等. 克罗伊茨费尔特-雅各布病的脑电图与临床所见8例报告[J]. 临床神经电生理学杂志, 2008, (3): 145-147. |
| 2008/4/30 | 中风与神经疾病杂志 | 戴晓蓉;邬剑军;董强; | [45]戴晓蓉,邬剑军,董强. Creutzfeldt-Jakob病11例临床分析[J]. 中风与神经疾病杂志, 2008, (2): 208-210. |
| 2008/8/27 | 中国神经精神疾病杂志 | 邱力;吴琪;方莹莹;黎锦如; | [46]邱力,吴琪,方莹莹,等. 克-雅氏病的临床特征与诊断(附2例报告)[J]. 中国神经精神疾病杂志, 2008, (8): 500-501. |
| 2006/4/1 | 吉林大学博士学位 | 左秀美 | [47]左秀美. 克—雅氏病新的潜在标志物H-FABP的检测及朊蛋白基因研究[D]. 吉林大学, 2006. |
| 2006/2/15 | 中华神经医学杂志 | 张家堂;蒲传强;贾渭泉;吴卫平;黄德辉;田成林;黄旭升;于生元;郎森阳; | [48]张家堂,蒲传强,贾渭泉,等. Creutzfeldt-Jakob病磁共振弥散加权像与临床表现及脑电图一致性的研究[J]. 中华神经医学杂志, 2006, (2): 188-191. |
| 2006/10/25 | 临床神经病学杂志 | 杜俊秋;徐建洋;王守勇; | [49]杜俊秋,徐建洋,王守勇. 散发性Creutzfeldt-Jakob病1例报告[J]. 临床神经病学杂志, 2006, (5): 375. |
| 2006/5/30 | 中国人兽共患病学报 | 孙岩;陈眉;张伟骏;张俐; | [50]孙岩,陈眉,张伟骏,等. Creutzfeldt-Jakob病临床及脑电图诊断分析[J]. 中国人兽共患病学报, 2006, (5): 466, 479-480. |
| 2006/5/15 | 中国神经免疫学和神经病学杂志 | 钱海蓉;戚晓昆;王巍;熊斌;王鲁宁; | [51]钱海蓉,戚晓昆,王巍,等. 散发型Creutzfeldt-Jakob病的临床、病理及头颅核磁DWI影像特点研究[J]. 中国神经免疫学和神经病学杂志, 2006, (3): 156-159, 198. |
| 2005/4/1 | 吉林大学博士论文 | 南善姬 | [52]南善姬. 克-雅氏病临床、病理、朊蛋白及其基因和蛋白质组学研究[D].吉林大学, 2005. |
| 2005/6/30 | 吉林大学博士学位论文 | 高金立 | [53]高金立. 脑脊液和血清s100β蛋白检测及s100β基因表达对克-雅氏病诊断价值的研究[D]. 吉林大学, 2005. |
| 2005/6/30 | 中华神经医学杂志 | 石强,黄旭升,田成林,于生元,管维平,吴卫平,蒲传强 | [54]石强,黄旭升,田成林,于生元,管维平,吴卫平,蒲传强. 克-亚二氏病的周围神经损害[J]. 中华神经医学杂志, 2005, (6): 599-601. |
| 2005/4/20 | 神经疾病与精神卫生 | 胡伟东,邵国富 | [55]胡伟东,邵国富. Creutzfeldt-Jakob病临床诊断1例分析[J]. 神经疾病与精神卫生, 2005, (2): 161-164. |
| 2005/12/23 | 中华神经医学杂志 | 关鸿志;郭玉璞;王珉珊;陈琳;刘秀琴;高晶; | [56]关鸿志,郭玉璞,王珉珊,等. 以视觉症状为主要表现的Creutzfeldt-Jakob病一例临床和病理[J]. 中华神经科杂志, 2005, (12): 763, 768. |
| 2004/3/25 | 郴州医学高等专科学校学报 | 李海鹏 | [57]李海鹏. Creutzfeldt-Jakob病(附一例临床和病理报告)[J]. 郴州医学高等专科学校学报, 2004, (1): 37-39. |
| 2004/10/30 | 中国康复医学杂志 | 林玉兰,叶小虹,黄莘莘,麦训良,罗小翠 | [58]林玉兰,叶小虹,黄莘莘,麦训良,罗小翠. Creutzfeldt-Jakob病与脑电图的关系[J]. 中国康复医学杂志, 2004, (10): 63. |
| 2003/9/20 | 医学研究生学报 | 周国庆,程茅薇,陈光辉 | [59]周国庆,程茅薇,陈光辉. 临床诊断的克罗伊茨费尔特-雅各布病[J]. 医学研究生学报, 2003, (9): 719. |
| 2003/2/23 | 中华神经科杂志 | 赵节绪,林世和,南善姬,江新梅,宋晓南 | [60]赵节绪,林世和,南善姬,江新梅,宋晓南. 散发性克-雅病PrP基因129密码子基因型与临床表型14例研究[J]. 中华神经科杂志, 2003, (1): 57-59. |
| 2002/2/26 | 卒中与神经疾病 | 赵伟秦,王得新,赵亚明,李继梅,许春玲,夏谦 | [61]赵伟秦,王得新,赵亚明,李继梅,许春玲,夏谦. Creutzfeldt-Jakob病5例临床研究[J]. 卒中与神经疾病, 2002, (1): 38-41. |
| 2001/7/20 | 中国医学影像技术 | 卢文甫,王鲁宁,蒲传强,王锦玲,王炜,朱明伟,汤洪川,于慧玲 | [62]卢文甫,王鲁宁,蒲传强,王锦玲,王炜,朱明伟,汤洪川,于慧玲. 国人Creutzfeldt-Jakob病的特征[J]. 中国医学影像技术, 2001, (7): 623-625. |
| 2001/3/30 | 中国实用内科杂志 | 曲方,于如山,张广生,徐惠琴,何祥,王耀山 | [63]曲方,于如山,张广生,徐惠琴,何祥,王耀山. Creutzfeldt-Jakob病8例临床诊断分析[J]. 中国实用内科杂志, 2001, (3): 159-160. |
| 1998/9/30 | 中国神经免疫学和神经病学杂志 | 江新梅,林世和,北本哲之,赵节绪,宋小南 | [64]江新梅,林世和,北本哲之,赵节绪,宋小南. 国人Creutzfeldt-Jakob病PrP基因表达变化的研究[J]. 中国神经免疫学和神经病学杂志, 1998, (3): 134-137. |
| 1994/9/15 | 中国神经免疫学和神经病学杂志 | 陈怀红，程源深，张宝荣 | [65]陈怀红，程源深，张宝荣. Creutzfeldt-Jakob病临床病理分析[J]. 中国神经免疫学和神经病学杂志, 1994, (2): 113-116. |
| 1993/12/27 | 陕西医学杂志 | 骆裕民,陈慧玲,关荷祥,徐祝华 | [66]骆裕民,陈慧玲,关荷祥,徐祝华. Creutzfeldt—Jakob病1例临床病理报告[J]. 陕西医学杂志, 1993, (12): 2, 41. |
| 1992/5/30 | 天津医药 | 王为民;裴世澄;温漓潮;卜积康; | [67]王为民,裴世澄,温漓潮,等. 脑组织活检对亚急性海绵状脑病生前确诊的重要意义(附四例临床病理报告)[J]. 天津医药, 1992, (5): 259-261, 320. |
| 1988/6/29 | 中国神经精神疾病杂志 | 赵明伦,韩仲岩,李净,张洪业 | [68]赵明伦,韩仲岩,李净,张洪业. Creutzfeldt-Jakob病(附1例临床病理报告)[J]. 中国神经精神疾病杂志, 1988, (3): 175-190. |
| 1986/5/1 | 中国神经精神疾病杂志 | 涂来慧,周广智,金克箕,丁素菊,戴益民,丛文铭 | [69]涂来慧,周广智,金克箕,丁素菊,戴益民,丛文铭. Creutzfeldt-Jakob病(附1例临床病理报告)[J]. 中国神经精神疾病杂志, 1986, (2): 116-117, 129. |
|  |  |  |  |
| 2019/3/27 | J Clin Neurosci | Kang, Y. J. | [70]Kang, Y. J., et al. (2019). "Diffusion-weighted imaging negative M232R familial Creutzfeldt-Jakob disease." J Clin Neurosci 64: 47-49. |
| 2009/12/24 | Acta Neurol Scand | Liu, Z. | [71]Liu, Z., et al. (2010). "Creutzfeldt-Jakob disease with PRNP G114V mutation in a Chinese family." Acta Neurol Scand 121(6): 377-383. |
| 2013/10/12 | Clin EEG Neurosci | Lv, Y. | [72]Lv, Y., et al. (2014). "A patient with progressive cognitive decline and periodic abnormal waves in EEG: PLEDs of neurosyphilis or PSDs of Creutzfeldt-Jakob disease?" Clin EEG Neurosci 45(3): 218-221. |
| 2011/5/21 | Prion | Shi, Q. | [73]Shi, Q., et al. (2011). "A Chinese Creutzfeldt-Jakob disease patient with E196K mutation in PRNP." Prion 5(2): 117-120. |
| 2013/6/15 | Prion | Shi, Q. | [74]Shi, Q., et al. (2013). "Rare V203I mutation in the PRNP gene of a Chinese patient with Creutzfeldt-Jakob disease." Prion 7(3): 259-262. |
| 2017/10/1 | Exp Ther Med | Wang, X. | [75]Wang, X., et al. (2017). "Three sporadic cases of Creutzfeldt-Jakob disease in China and their clinical analysis." Exp Ther Med 14(3): 2664-2670. |
| 2018/1/10 | Medicine (Baltimore) | Yao, Y. | [76]Yao, Y., et al. (2017). "Cerebrospinal fluid real-time quaking-induced conversion test for sporadic Creutzfeldt-Jakob disease in an 18-year-old woman: A case report." Medicine (Baltimore) 96(48): e8699. |
| 2015/1/4 | Cell Biochem Biophys | Zhao, X. | [77]Zhao, X., et al. (2015). "Comparison Between Sporadic and Misdiagnosed Sporadic Creutzfeldt-Jakob Disease: A Report of Two Cases." Cell Biochem Biophys 72(2): 311-315. |
| 2010/8/23 | Biomedical and environmental sciences : BES | Gao C | [78]C, G., et al. (2010). "The first Chinese case of Creutzfeldt-Jakob disease with mutation of E200K in PRNP." Biomedical and environmental sciences : BES 23(2): 158-160. |
|  |  |  |  |
